# On the optimal temporal resolution for phase contrast cardiovascular magnetic resonance imaging: establishment of baseline values

**DOI:** 10.1186/s12968-020-00669-1

**Published:** 2020-10-05

**Authors:** Francesco Santini, Michele Pansini, Maja Hrabak-Paar, Denise Yates, Thomas H. Langenickel, Jens Bremerich, Oliver Bieri, Tilman Schubert

**Affiliations:** 1 Department of Radiology, Division of Radiological Physics, University Hospital Basel, Petersgraben 4, 4031 Basel, Switzerland; 2 Department of Biomedical Engineering, University of Basel, Allschwil, Switzerland; 3 Ricerche Diagnostiche Srl, Bari, Italy; 4 University Hospital Center Zagreb, University of Zagreb School of Medicine, Zagreb, Croatia; 5 Novartis Institutes of Biomedical Research, Cambridge, MA USA; 6 Novartis Institutes for Biomedical Research, Translational Medicine, Basel, Switzerland; 7 Ethris GmbH, Planegg, Germany; 8 Department of Radiology, University Hospital Basel, Basel, Switzerland; 9 Department of Neuroradiology, Zurich University Hospital, Zurich, Switzerland

**Keywords:** Phase contrast MRI, Doppler ultrasound, Frequency content, Temporal resolution

## Abstract

**Background:**

The aim of this study is to quantify the frequency content of the blood velocity waveform in different body regions by means of phase contrast (PC) cardiovascular magnetic resonance (CMR) and Doppler ultrasound. The highest frequency component of the spectrum is inversely proportional to the ideal temporal resolution to be used for the acquisition of flow-sensitive imaging (Shannon-Nyquist theorem).

**Methods:**

Ten healthy subjects (median age 33y, range 24–40) were scanned with a high-temporal-resolution PC-CMR and with Doppler ultrasound on three body regions (carotid arteries, aorta and femoral arteries). Furthermore, 111 patients (median age 61y) with mild to moderate arterial hypertension and 58 patients with aortic aregurgitation, atrial septal defect, or repaired tetralogy of Fallot underwent aortic CMR scanning. The frequency power distribution was calculated for each location and the maximum frequency component, f_max_, was extracted and expected limits for the general population were inferred.

**Results:**

In the healthy subject cohort, significantly different f_max_ values were found across the different body locations, but they were nonsignificant across modalities. No significant correlation was found with heart rate. The measured f_max_ ranged from 7.7 ± 1.1 Hz in the ascending aorta, up to 12.3 ± 5.1 Hz in the femoral artery (considering PC-CMR data). The calculated upper boundary for the general population ranged from 11.0 Hz to 27.5 Hz, corresponding to optimal temporal resolutions of 45 ms and 18 ms, respectively. The patient cohort exhibited similar values for the frequencies in the aorta, with no correlation between blood pressure and frequency content.

**Conclusions:**

The temporal resolution of PC-CMR acquisitions can be adapted based on the scanned body region and in the adult population, should approach approximately 20 ms in the peripheral arteries and 40 ms in the aorta.

**Trial registration:**

This study presents results from a restrospective analysis of the clinical study NCT01870739 (ClinicalTrials.gov).

## Background

Quantitative phase contrast (PC) cardiovascular magnetic resonance Imaging (CMR) is a widely used clinical application to measure flow volume and velocity. Furthermore, the method has been proven to be a robust and reliable tool to measure flow independently of the anatomic localization [1, 2]. Even though PC-CMR is regarded as a user-independent tool, errors may arise in consequence of insufficient spatial or temporal resolution as well as inappropriate velocity encoding methods [3–5]. More recently, advanced PC-CMR methods have been developed which allow visualization of previously unavailable information, in particular with respect to three-dimensional, three-directional encoding [6–8]. Optimization of the scanning protocol has generated a considerable lengthening of acquisition time and an approach has been developed to streamline the settings to evaluate blood flow velocity.

A usual tradeoff for a shorter scan time is reducing the temporal resolution of the acquisition by acquiring a higher number of k-space lines for each cardiac phase. Although routine practice has shown that too low temporal resolution leads to inaccurate results, little data exist regarding the optimal temporal resolution of PC-CMR [5]. The existing evidence is based on the accuracy of the extracted parameters of the flow curves rather than spectral content of the curve itself [9]. Here, an evidence-based method, providing the required information to setup the optimal temporal resolution for the signal acquisition would be highly advisable to acquire a correct velocity waveform in the minimum time without losing important information of the signal dynamics.

The optimal temporal resolution for the sampling of a continuous signal is given by the Nyquist-Shannon theorem, a fundamental theorem in digital signal processing, which defines as optimal the inverse of twice the highest frequency of the spectrum of the signal (Nyquist rate).

The purpose of the present study was to prospectively investigate the frequency content of the velocity waveform in order to identify the optimal temporal resolution for PC-CMR. Therefore, we cross-validated oversampled PC-CMR with Doppler ultrasound in healthy subjects to ensure that no valuable portion of the frequency spectrum was lost in the PC encoding. In a second step, we analyzed oversampled CMR data from a cohort of patients suffering from arterial hypertension, in order to determine whether presence of disease leads to a modified spectral content compared to healthy subjects. Finally, we validated the derived temporal resolution thresholds in a second patient cohort.

## Methods

### Study population – healthy cohort

Ten healthy subjects with no known significant health problems (median age 33y, range 24–40) were included in the first part of the study. Each subject underwent a CMR examination and, in a separate session, a Doppler ultrasound examination. The study was performed in compliance with local ethics regulations.

### Study population – patient cohort

Baseline data from a cohort of 111 patients with mild to moderate arterial hypertension (median age 61y, range 23–80) were retrospectively analyzed. These patients were participating in a clinical study (ClinicalTrials.gov Identifier: NCT01870739) performed at three different centers [10]. The clinical trial was performed in compliance with health authority approval and local ethics regulations. Brachial arterial pressure was measured and central mean pressure (CMP) was estimated using applanation tonometry [11].

### Study population – patient validation cohorts

In order to validate the recommendations obtained from the previous two cohorts, a retrospective validation study on clinical flow acquisitions in multiple pathologies was performed. Patients who had a PC-CMR scan including the ascending and descending aorta or including the aortic valve were retrospectively selected from the routine examinations over a period of 3 years. The patient reports were examined and hemodynamic-relevant pathologies were selected for subsequent evaluation. The following patients were selected:

- 42 patients with the clinical question of aortic regurgitation (flow measurement through the aortic valve), median age 57y, range 19–79;
- 7 patients with confirmed atrial septal defect (ASD, flow measurement in the ascending aorta (AAo) and descending aorta (DAo)), median age 63y, range 19–63;
- 9 patients with repaired tetralogy of Fallot (flow measurement in the AAo and DAo), median age 29y, range 21–43.

### CMR examination

#### Healthy cohort

All CMR examinations were performed on a 3 T whole-body CMR scanner (MAGNETOM Prisma, Siemens Healthineers, Erlangen, Germany). Each subject was prepared with a head and neck receive coil (24 channels) and two surface body arrays (18 channels each) covering the thorax and the pelvic region. A spine coil (32 channels) was integrated into the table. A pulse oximeter was attached to the index finger of the right hand to obtain the photoplethysmogram for cardiac gating.

Retrospectively-gated PC-CMR images were obtained in transversal orientation at three different body locations: at the common carotid artery (CCA), proximally with respect to the carotid bifurcation, at the ascending and descending aorta at the level of the pulmonary artery bifurcation, and at the common femoral artery (CFA), proximally with respect to the branching of the profunda femoris artery.

The sequence was a single-slice PC radiofrequency-spoiled gradient echo with a flip angle of 20° and a receive bandwidth of 620 Hz/px. Other parameters were adapted according to the scanned location and are summarized in Table 1. The temporal resolution in the aorta was half with respect to the periphery because of the necessity for breath-holding during image acquisition. Velocity encoding was through-plane and the velocity encoding (Venc) was set to 150 cm/s for all locations. The heart rate was recorded during the scan in the form of average RR interval in milliseconds.

**Table 1:** Summary of CMR sequence parameters at different locations

Location	Resolution (mm^3^)	Matrix size	Actual temporal resolution (ms)	TR/TE (ms)	Reconstructed cardiac phases
CCA	1x1x4	192x144x1	10	5/2.9	100
Ao	2.7 × 2.7 × 6	128x79x1	20	5/2.5	100
CFA	1.25 × 1.25 × 4	256x176x1	10	5/2.9	100
Ao/Validation	1.9 × 1.9 × 6	208x144x1	40	5/2.7	30

*CCA* Common carotid artery, *Ao* Aorta, *CFA* Common femoral artery, *TR* Repetition time, *TE* Echo time

#### Patient cohort

All scans were performed on 3 T CMR scanners (MAGNETOM Skyra or Prisma, Siemens Healthineers) with the same CMR protocol. Retrospectively electrocardiography-gated PC-CMR images were obtained in transversal orientation at the AAo and DAo at the level of the pulmonary artery.

The sequence was a single-slice PC radiofrequency-spoiled gradient echo with the same parameters as for the healthy cohort.

#### Patient validation cohort

The scans for these patients were executed during conventional diagnostic examinations with a standard clinical protocol on a 1.5 T CMR scanner (MAGNETOM Avanto Fit, Siemens Healthineers). The used sequence had a lower temporal resolution but a higher spatial resolution than the one used for the first part of the study, however, temporal resolution was within the threshold derived from the healthy subject and patient study described below. The sequence type was a single-slice phase-contrast radiofrequency-spoiled gradient echo. The relevant sequence parameters are given in Table 1.

### Doppler ultrasound

Doppler ultrasound measurements were acquired for the healthy subject cohort only. Doppler ultrasound examinations were performed on a state-of-the-art ultrasound scanner (Aplio 500, Toshiba Medical Systems Corp, Tochigi, Japan) equipped with a 12 MHz vascular probe. All Doppler ultrasound examinations were performed by the same radiologist with 6 years of experience in vascular ultrasound. Examinations were conducted according to the American Institute of Ultrasound in Medicine practice guidelines [12, 13].

Doppler signal was obtained bilaterally in the CCAs (2–3 cm below the bifurcation) and in the CFAs, with the angle between the direction of flowing blood and the applied Doppler ultrasound signal not exceeding 60°. The envelope detection of the ultrasound system was used to extract the velocity signal with a temporal resolution of 2 ms.

### CMR signal analysis

All retrospectively-gated CMR images were reconstructed using the method provided by the scanner manufacturer, which implements linear interpolation. According to [14], this introduces low-pass filtering with cutoff frequencies of 44 Hz (periphery) and 22 Hz (aorta) of the velocity signal.

The velocity waveform was extracted by drawing a region-of-interest on each vessel (left and right CCA (healthy subjects), left and right CFA (healthy subjects) and AAo and DAo (healthy subjects and patients)) and averaging the phase signal over the vessel surface for each cardiac phase.

The velocity signal was mean-detrended to eliminate the bulk-flow contribution to the spectrum and zero-padded to 1000 samples to increase the number of points of the subsequent Fourier transform. The power spectrum was calculated by taking the squared magnitude of the discrete Fourier transform (DFT) of the signal:

$$ P(f)={\left| DFT\left(v(t)\right)\right|}^2, $$

where P is the power, *f* the frequency, and *v* is the zero-padded mean-detrended velocity signal. The frequency below which 95% or 99% of the total signal energy was contained was considered as the highest spectral content and indicated as f_max95_ and f_max99_, so that

$$ \underset{0}{\overset{f_{\max \left\{95,99\right\}}}{\int }}P(f) df=\left\{\mathrm{0.95,0.99}\right\}\underset{0}{\overset{1/2T}{\int }}P(f) df, $$

where T is the temporal resolution of the acquisition (and therefore 1/2*T* is the maximum measurable frequency of the spectrum).

Said f_max_ was calculated for each location of each healthy subject (for a total of 20 values in the CCA, 20 in the CFA, 10 in the AAo and 10 in the DAo), and in the aorta of each patient.

### Statistical evaluation

The characteristics of the statistical distribution of maximum frequancy component (f_max_) values were studied by extracting the average and standard deviation from each location across all the subjects. The upper boundary of the distribution was defined by summing three times the measured standard deviation to the measured average, in order to identify a value within which 99.7% of the population would be contained. The minimum sampling rate associated to the upper boundary (and defined as 1/(2*f*_max_)), and therefore with good approximation to the general population, was calculated for each location and modality.

Inferential statistics was applied to the values of the healthy subject cohort in order to study the significance of differences across different locations and modalities. To this end, a linear mixed effects model was applied to the data, using location (AAo as reference) and acquisition modality as fixed effects and subject and laterality (nested within subject) as random effects for which separate intercepts were fit. Analysis of variance (ANOVA) test was used to assess the significance of the fixed effects. A *p*-value of 0.05 or lower was considered statistically significant.

Pearson’s correlation coefficient r between RR interval and cutoff frequency was calculated globally and for each location. *P*-value was derived from the coefficient and the significance threshold was considered 0.05. In the case of location-based analysis, a Bonferroni correction for multiple comparisons was applied, thus lowering the significance threshold to 0.0125.

In patients, Pearson’s correlation was calculated between systolic and diastolic pressure and calculated spectral content, as well as age.

Finally, a student’s t-test was used to assess differences between frequencies measured in healthy subjecs and patients.

All statistical analyses were performed using the software package R [15] (R Foundation for Statistical Computing, Vienna, Austria) with the additional package lme4 [16].

## Results

### Healthy subject cohort

**Fig. 1:**
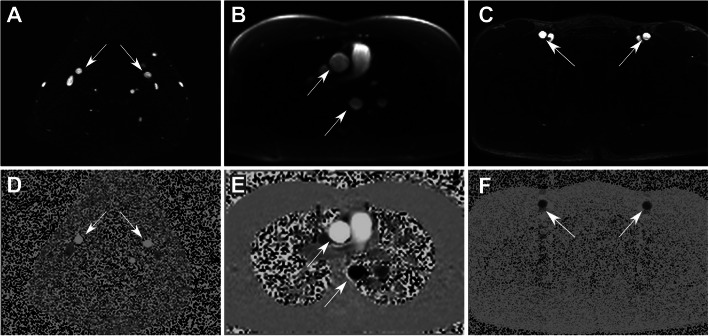
Exemplary CMR images at the three locations: common carotid artery (**a**, **d**); aorta (**b**, **e**); common femoral artery (**c**, **f**). The top row represents magnitude images, and the bottom row represents phase contrast images. Arrows point at the vessels of interest

**Fig. 2:**
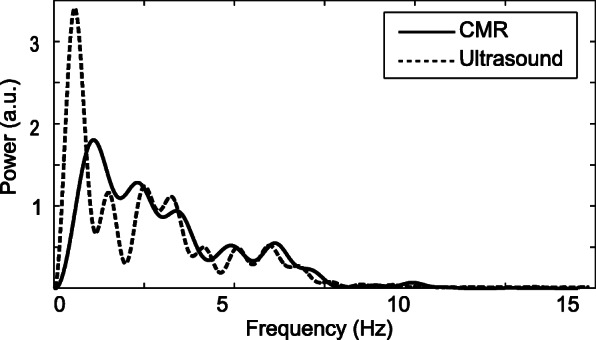
Spectrum of one waveform acquired at the same location by CMR (solid line) and Doppler (dashed line)

Representative CMR images obtained at the three scanned locations are shown in Fig. 1. The CMR and Doppler signals resulted in visually similar power spectra, especially in terms of maximum frequency content (Fig. 2).

**Fig. 3:**
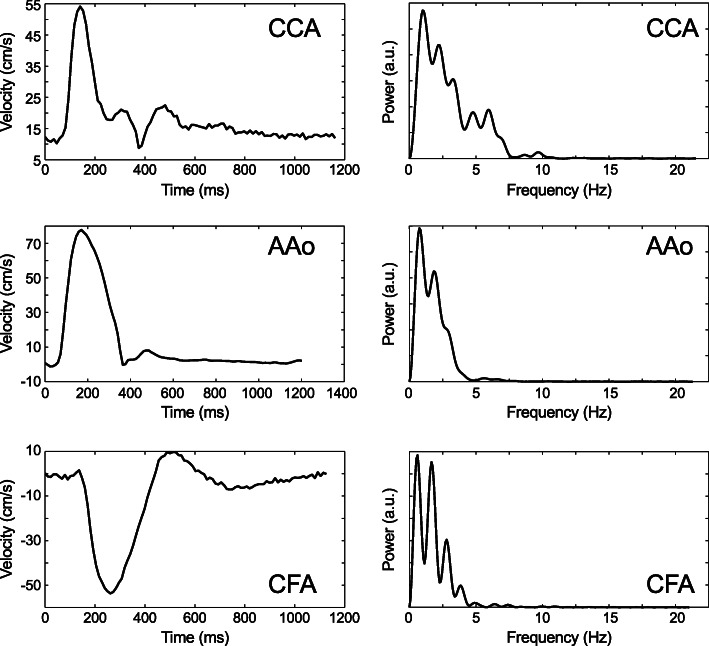
Velocity waveforms (left) and corresponding power spectra (right) at three different locations (CCA = common carotid artery, AAo = ascending aorta, CFA = common femoral artery) in one healthy subject

The spectra at the level of the CCA were consistently higher than the spectra of the aortic waveforms, whereas the CFA exhibited a much higher variability. Representative velocity waveforms and corresponding spectra are shown in Fig. 3.

**Fig. 4:**
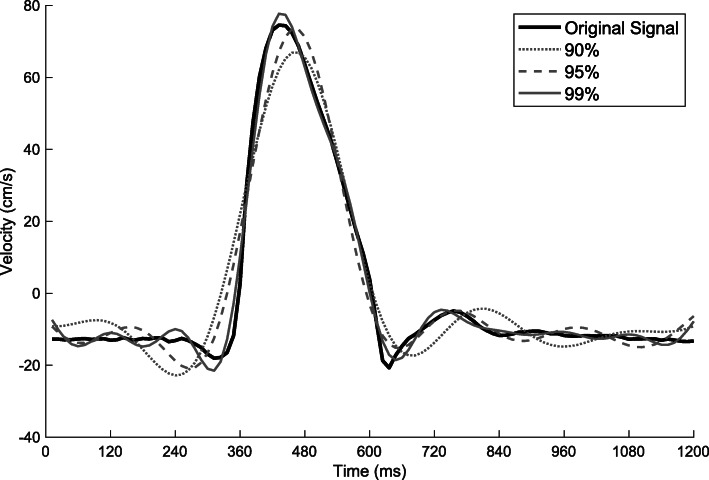
Representative (mean-detrended) velocity waveform in the ascending aorta of a healthy subject reconstructed from a full spectrum (solid black line) and with various percentages of the spectrum retained (99, 95, and 90%)

**Fig. 5:**
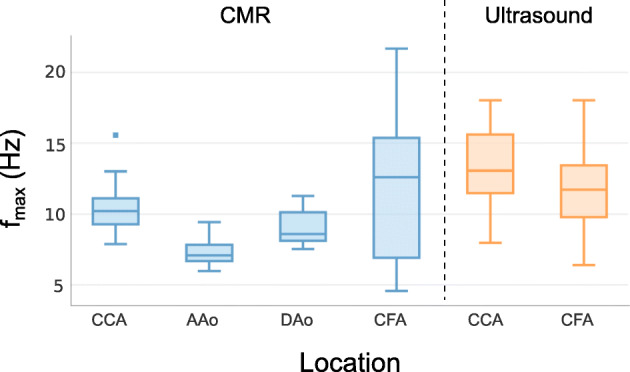
Distributions of maximum detected frequencies across the healthy subjects grouped by location and modality (CCA = Common Carotid Artery, AAo = Ascending Aorta, DAo = Descending Aorta, CFA = Common Femoral Artery)

Retaining 99% of the spectral components resulted in better fidelity of the depiction of the reconstructed waveform, whereas a 95% limit still seems to reasonably capture the peak velocity but the rise time of the velocity waveform is compromised. A representative flow waveform at different percentages of spectral components is shown in Fig. 4. The subsequent inferential statistical evaluations refer to a 99% spectral cutoff value, as it is the one that best describes the flow waveform.

f_max_ statistics are summarized in Table 2 and visually represented in Fig. 5. The upper boundaries of the f_max99_ values ranged from 11 Hz in the AAo to 27 Hz in the CFA, resulting in recommended sampling rates ranging from 18 ms in the CFA to 45 ms in the AAo; when using the lower cutoff percentage of 95%, the boundaries ranged from 4.9 Hz in the AAo to 10.4 Hz in the CCA, corresponding to recommended sampling rates of 48 ms to 103 ms.

**Table 2:** Summary of the descriptive statistics for f_max_ in healthy subjects at different locations and measured by different modalities, for two cutoff values of spectral energy (95% and 99%). The upper boundary is defined as the mean plus three times the standard deviation and it is the value below which 99.7% of the population is contained

Modality	Location	95%				99%			
		f_**max95**_ (Hz)			Nyquist rate (ms)	f_**max99**_ (Hz)			Nyquist rate (ms)
		Mean	SD	Upper boundary		Mean	SD	Upper boundary	
**CMR**	**CCA**	6.8	0.4	8.0	62	10.7	1.7	15.8	32
	**CFA**	4.5	0.7	6.6	75	12.3	5.1	27.5	18
	**AAo**	3.9	0.3	4.9	103	7.7	1.1	11.0	45
	**DAo**	3.9	0.3	4.9	103	9.3	1.2	13.0	38
**US**	**CCA**	7.1	1.1	10.4	48	13.6	2.8	21.9	22
	**CFA**	4.9	0.9	7.6	66	12.0	3.1	21.4	23

*CCA* Common carotid artery, *CFA* Common femoral artery, *AAo* Ascending aorta, *DAo* Descending aorta, *SD* Standard deviation, *CMR* Cardiovascular magnetic resonance, *US* Ultrasound

The linear mixed effects model resulted in negligible variances explained by the random effects (subject and laterality), whereas, for the fixed effects, the f_max99_ variable showed highly significant differences as a function of location (*p* < 0.01) and non-significant differences with respect to modality (*p* = 0.06).

The f_max99_ variable showed no significant correlation with the duration of the heart cycle (median heart cycle duration across the subjects 942.5 ms, range 900–1220), neither globally (*p* = 0.55), nor in any location (*p*-values ranging from 0.03 to 0.38, compared with a corrected significance level of 0.0125).

### Patient cohort

The population presented mean f_max99_ of 9.3 ± 1.4 Hz in the AAo, and 8.6 ± 1.4 Hz in the Dao. The two results are significantly different (*p* < 0.001) and led to an upper boundary of 13.7 Hz in the AAo and 12.9 Hz in the DAo, respectively. The optimal temporal resolution for a flow measurement acquisition still able to capture the whole frequency content would therefore be 36 ms and 39 ms respectively.

The f_max95_ was 5.4 ± 0.7 Hz in the AAo and 4.9 ± 0.8 Hz in the DAo, resulting in optimal temporal resolutions of 67 ms and 68 ms respectively.

The spread of brachial pressures across the population was 135 ± 19 mmHg (systolic, range 95–210) and 80 ± 13 mmHg (diastolic, range 41–116).

**Fig. 6:**
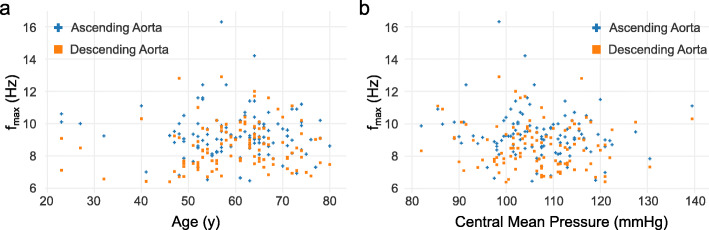
f_max99_ distributions in the ascending aorta (blue) and descending (orange) aorta with respect to patient age (**a**) and estimated central mean pressure (**b**)

The f_max99_ in the AAo and DAo showed no correlation with age or central mean pressure (see Fig. 6).

Between the healthy subject and patient population, the difference in f_max99_ was significantly different in the AAo (*p* < 0.01), but not in the Dao (*p* = 0.12).

### Validation cohort

No patient in the validation cohort had f_max99_ values higher than the proposed respective limits, and the used temporal resolution of 40 ms (corresponding to a Nyquist frequency of 12.5 Hz) was sufficient in all cases. Specifically, the flow through the aortic valve in the aortic regurgitation population showed a range of f_max99_ between 4.2 Hz and 10.5 Hz (median 7.1 Hz); the ASD population showed a range of 6.2 Hz to 9.5 Hz (median 7.0 Hz) in the AAo and 6.3 Hz to 8.2 Hz (median 7.25 Hz) in the DAo; the repaired tetralogy of Fallot population presented a range of 4.9 Hz to 10.8 Hz (median 7.1 Hz) in the AAo and 5.7 Hz to 9.2 Hz (median 6.4 Hz) in the DAo. Regarding the distribution of f_max95_, the predicted upper boundary of 4.9 Hz was exceeded by: 5 out of 42 (12%) aortic insufficiency patients (median 3.8 Hz, range 3.0–6.6), 1 out of 7 (14%) ASD patients (median 4.4 Hz, range 3.2–5.1), and 1 out of 9 (11%) tetralogy of Fallot patients (median 3.8 Hz, range 3.1–5.8).

## Discussion

In this study, we characterized the frequency content of the velocity waveform in order to identify the optimal temporal resolution for the acquisition of PC-CMR data. We were able to show that PC-CMR can be performed on state-of-the art scanners with sufficiently high temporal resolution to capture the maximum frequency content. Furthermore, we showed that the aortic frequency content between healthy young adults and older patients with mild hypertension is significantly different. We found a lower frequency content in the aorta compared to the femoral and common carotid arteries. These findings imply that different temporal resolutions should be applied for different body regions. In large, central vessels, low frequencies dominate, making it possible to sample with a lower temporal resolution, whereas in peripheral vessels this must be increased as higher frequencies prevail.

Our results show that in all cases the chosen PC-CMR temporal resolution was sufficient to capture the signal components, the highest measured frequencies overall being approximately 20 Hz, corresponding to a required temporal resolution of 25 ms.

This finding is impactful, because too low temporal resolution leads to inaccurate results since the high frequencies cannot be properly sampled, and also an unessential high temporal resolution results in a needless scan time increase.

We performed our analysis by assuming that a waveform would be “correctly” sampled if either 95% or 99% of its spectrum was retained. In some cases, either assumption can be valid and justified. However, we have observed that while a 95% cutoff value can generally correctly depict the peak velocity, the correct depiction of the flow acceleration requires 99% of the spectrum to be represented, which can be crucial in the evaluation of derived parameters as, for example, pulse wave velocity. The distribution of the f_max99_ values showed larger variability than f_max95_; this is most likely due to the noise, which dominates the high frequencies. However, this variability leads to more conservative results for the upper boundary of the distribution, and the results in the validation cohort showed that the inferred values for f_max99_ are still valid in a larger number of cases and conditions, as no subject exceeded the predicted threshold.

Additionally, we demonstrated that frequencies in the aorta were significantly lower than those evaluated in the periphery. This finding seems to contradict the classic Windkessel effect used for vascular modeling, where the vessel structure should provide a dampening effect and therefore a low-pass filtering in the frequency domain. Our results can, however, be explained by the nonlinear nature of the system, and the contribution of the reflected wave becoming more prominent in peripheral vessels, thus generating higher-frequency contributions. Also interesting to note, the f_max_ values do not significantly depend on the heart rate. The explanation is likely that changes in the heart rate only affect the diastolic phase, when the flow is approximately constant and does not contain high frequency components. This finding is useful because it allows the definition of more general, non-patient-specific protocols.

The hypertensive patient data showed higher frequencies in the ascending and descending aorta compared to healthy subjects. This is in accordance with the finding that the arterial wall stiffens with age and leads to a lower compliance [17]. However, the acquired temporal resolution in this study was sufficient to sample the frequency content in the patient cohort, being almost twice as high (20 ms) as the resultant optimal temporal resolution in the AAo (36 ms) and DAo (39 ms) for f_max99_. No dependency of spectral component on arterial pressure was found in the patient cohort.

The results of our study are in accordance with existing data. Holdsworth et al. [18] investigated the physiological velocity waveforms in humans in the frequency domain and identified frequencies in the carotid arteries of healthy subjects up to 12 Hz using Doppler ultrasound. In our setup, two out of twenty data points showed frequencies higher than 12 Hz when using a 99% of energy as a cutoff value; when using a value of 95%, as in Holdsworth et al., our findings predict frequencies up to 10.4 Hz.

The findings of the present work may be used as a guideline for the definition of acquisition protocols based on PC-CMR. However, while sampling at the Nyquist rate guarantees that the information of the signal is retained in the process, a proper interpolation of the velocity signal is required in order to restore the complete signal characteristics (peak velocity, acceleration, etc.). The simple analysis of the tabular data might still lead to underestimation of some parameters, and a temporal interpolation in the signal processing sense (upsampling and low-pass filtering, or, similarly, bspline interpolation) is the preferred method. This is not usually implemented in the commercial flow analysis interfaces and might require additional postprocessing.

Another important consideration relates to spatio-temporal (so-called k-t) acceleration methods [19–21], and in general to other methods that involve interpolation. These methods exploit a temporal correlation among the signals. If the true “temporal footprint” (the temporal span that provides information of a single signal sample) is lower than the Nyquist rate, such correlation cannot be guaranteed and inaccuracies might arise. Therefore, we recommend avoiding high spatio-temporal accelerations unless the temporal footprint of the method is well known and the frequency characteristics of the method well evaluated.

To our knowledge, this is the first study to investigate the optimal temporal sampling resolution for PC-CMR by means of analyzing the frequency content of the flow waveform with CMR. Establishing reference values for PC-CMR is important, as this might lead to guidelines in the future that direct this increasingly used technique towards a fully reproducible, quantitative imaging technique. Based on our results, we recommend that the acquisition protocols should aim to sample the signal in a way that retains 99% of the spectrum, because this preserves peak velocities, accelerations, and gives more conservative limits in general. For this, a temporal resolution of 20 ms in the peripheral vessels, and of 40 ms in the aorta are recommended. If strict requirements of scan time and/or spatial resolution are in place, these temporal resolutions can be lowered to 50 ms and 100 ms respectively (corresponding to a 95% of the spectral content), with the knowledge that some signal dynamics will be lost in the acquisition.

## Conclusions

In this work, we objectively established the optimal temporal resolution for the acquisition of PC-CMR images in the aorta and large conduit arteries of the cranium and lower extremity in healthy young adults and hypertensive middle-aged individuals. The optimal temporal resolution depends on anatomic location. We could demonstrate that for the aorta, approximately 40 ms or lower is sufficient, while for peripheral conduit arteries (CFA and CCA) the temporal resolution should be set to approximately 20 ms or lower for optimal sampling to evaluate blood flow and velocity.

## Data Availability

Informed consent obtained does not allow further dissemination of clinical data.

## Abbreviations

AAo: Ascending aorta
ASD: Atrial septal defect
CCA: Common carotid artery
CFA: Common femoral artery
CMP: Central mean pressure
CMR: Cardiovascular magnetic resonance
DFT: Discrete Fourier transform
DAo: Descending aorta
f_max95_: Frequency below which 95% of the spectral energy is represented
f_max99_: Frequency below which 99% of the spectral energy is represented
PC: Phase contrast
